# Continuous Spatiotemporal Therapy of A Full-API Nanodrug via Multi-Step Tandem Endogenous Biosynthesis

**DOI:** 10.1038/s41467-023-37315-0

**Published:** 2023-03-25

**Authors:** Fang Fang, Sa Wang, Yueyue Song, Meng Sun, Wen-Cheng Chen, Dongxu Zhao, Jinfeng Zhang

**Affiliations:** 1 Key Laboratory of Molecular Medicine and Biotherapy, School of Life Science, Beijing Institute of Technology, Beijing, 100081 P.R. China; 2 School of Chemical Engineering and Light Industry, Guangdong University of Technology, Guangzhou, 510006 P.R. China

**Keywords:** Drug delivery, Drug development, Nanoparticles, Cancer microenvironment

## Abstract

Nanomedicine holds great promise to enhance cancer therapy. However, low active pharmaceutical ingredient (API) loading content, unpredictable drug release, and potential toxicity from excipients limit their translational capability. We herein report a full-API nanodrug composed of FDA-approved 5-aminolevulinic acid (ALA), human essential element Fe^3+^, and natural bioactive compound curcumin with an ideal API content and pH-responsive release profile for continuous spatiotemporal cancer therapy achieved by multi-step tandem endogenous biosynthesis. First, ALA enzymatically converts into photosensitizer protoporphyrin IX (PpIX). Afterward, multiple downstream products including carbon monoxide (CO), Fe^2+^, biliverdin (BV), and bilirubin (BR) are individually biosynthesized through the PpIX-heme-CO/Fe^2+^/BV-BR metabolic pathway, further cooperating with released Fe^3+^ and curcumin, ultimately eliciting mitochondria damage, membrane disruption, and intracytoplasmic injury. This work not only provides a paradigm for exploiting diversified metabolites for tumor suppression, but also presents a safe and efficient full-API nanodrug, facilitating the practical translation of nanodrugs.

## Abstract (web summary)

Nanomedicine is important in cancer therapy, but loading, drug release, and therapeutic effectiveness issues limit the translation to the clinic. Here, authors report a full-API nanodrug with an ideal API content and pH-responsive release for continuous spatiotemporal cancer therapy based on PpIX-heme-CO/Fe^2+^/BV-BR metabolic pathway.

## Introduction

With the booming development of nanotechnology, a number of nanodrugs such as anticancer formulation Doxil® and COVID-19 mRNA vaccine Comirnaty® with reduced side effects or enhanced therapeutic efficacy have received marketing approval, changing the landscape of conventional therapeutics^1–5^. However, there remain considerable challenges limiting the further clinical application of the nanoformulations, including the poor active pharmaceutical ingredient (API) loading content (typically <10 wt%), inferior biodegradability, potential toxicity, and immunogenicity stemming from the excipients, as well as tedious fabrication process^6,7^. Fortunately, the easy-to-fabricate carrier-free nanodrugs totally assembled from antitumor agents in absence of any exogenous inert carriers, as a promising subdiscipline of nanomedicine, could overcome the aforementioned dilemmas^8–14^. Nevertheless, their further developments are still impeded by i) undesired physiological stability due to the weak driving forces; ii) off-target adverse effects because of severe premature drug leakage; iii) compromised therapeutic effectiveness relying on limited therapeutic modalities. Thereby, developing a smart nanodrug with desirable API loading, site-specific release potency, and continuously multiple therapeutic mechanisms for cancer treatment is an urgent demand yet tricky task.

Inspired by old drugs such as remdesivir repurposing in COVID-19^15,16^, exploiting nanoformulations based on clinically approved drugs could simultaneously minimize the overall developmental costs and evaluation timelines, accelerating the practical translation of the nanoformulations^17–20^. However, among the approved drugs, either their subcellular organelle-targeted capabilities are lacking, or only a single intracellular site of action could be triggered by these drugs. Moreover, their therapeutic modalities are relatively limited, leading to the unsatisfied therapeutic efficiency and potential toxicity caused by the indispensable repeated administration. On these grounds, 5-aminolevulinic acid (ALA) as a US Food and Drug Administration (FDA)-approved prodrug draws our attention, which can endogenously biosynthesize red-fluorescent protoporphyrin IX (PpIX) for photodynamic therapy (PDT) by enzymatic reactions^21–26^. It is noteworthy that the PpIX downstream metabolites such as carbon monoxide (CO)^27–30^, Fe^2+ ^^31–35^, biliverdin (BV)^36–39^, and bilirubin (BR)^40,41^ produced during the heme metabolic pathway also show potential therapeutic bioactivities acting on different subcellular sites, which offers promising potential for synergistic cascade tumor therapy. Nonetheless, to the best of our knowledge, leveraging a series of endogenous PpIX downstream metabolites to boost collaborative tumor suppression has rarely been reported.

**Fig. 1:**
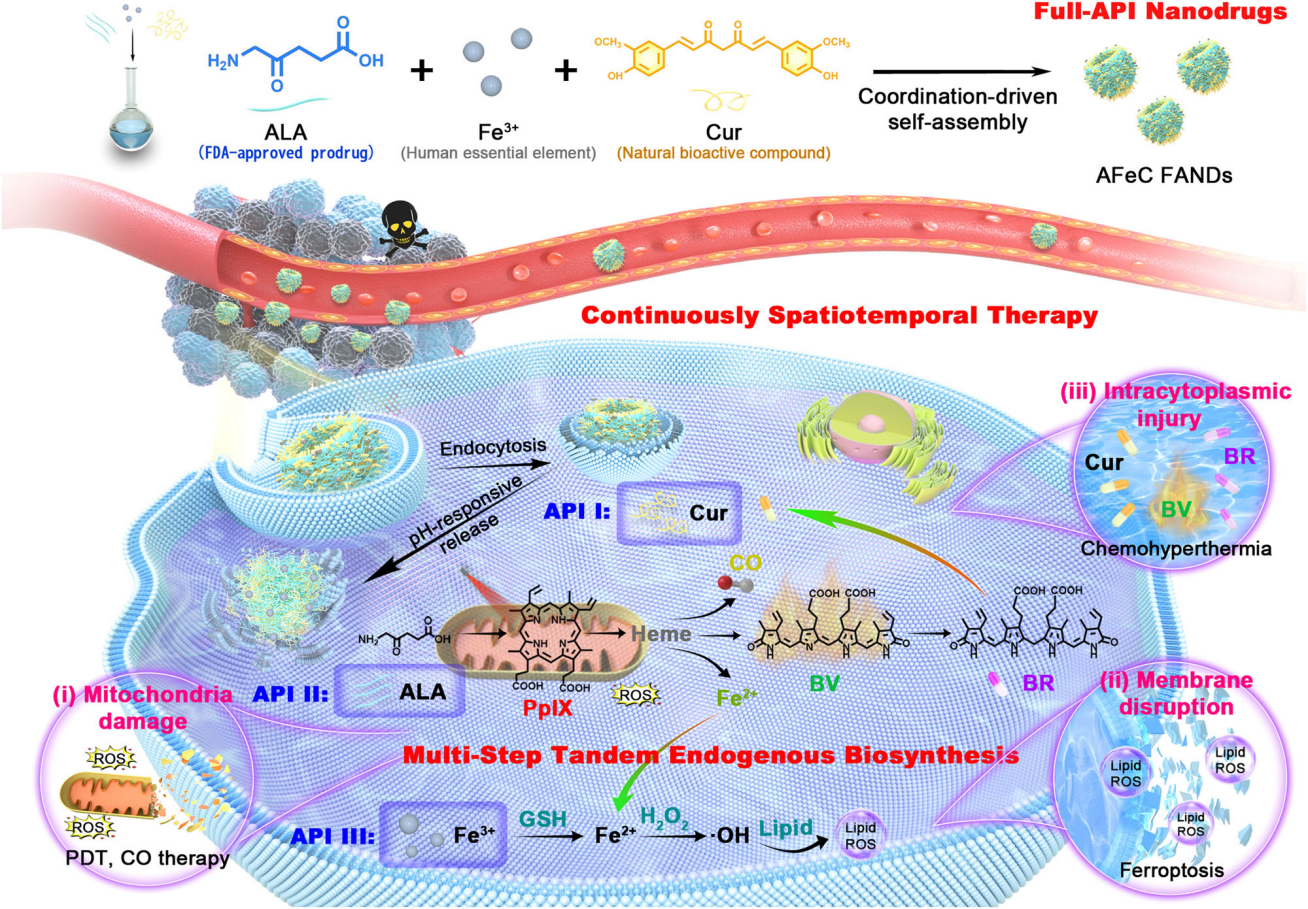
Schematic illustration of multi-step tandem endogenous biosynthesis triggered by the AFeC FANDs for continuous spatiotemporal cancer therapy. First, the Full-API nanodrugs consisted of FDA-approved ALA and Cur, as well as human essential Fe^3+^ were fabricated by the metal coordination-driven self-assembly, which could accumulate at tumor sites via the enhanced permeability and retention (EPR) effect, and release active ingredients in acid tumor cells. Importantly, the released ALA could endogenously biosynthesize multiple metabolic products including PpIX, carbon monoxide (CO), Fe^2+^, biliverdin (BV), and bilirubin (BR), which further synergized with the released Fe^3+^ and curcumin (Cur), ultimately inducing mitochondria damage, membrane disruption, and intracytoplasmic injury.

Herein, we design a full-API nanodrug (FAND) based on FDA-approved 5-aminolevulinic acid (ALA), human essential element Fe^3+^, and natural bioactive compound curcumin (Cur) (termed as AFeC FANDs) with an ideal API content of 100 wt% for continuous spatiotemporal therapy with only a single-dose administration at single-laser irradiation (Fig. 1). On one hand, besides weak non-covalent forces including π-π stacking, hydrogen bond, and hydrophobic interactions, the formation of the AFeC FANDs are also dominantly driven by metal-ligand coordination. Attractively, such synergistic interplay of multiple interactions greatly enhances their stability under physiological environments, as well as endows the FAND with a pH-responsive release feature in acid environments. On the other hand, continuous spatiotemporal therapy of the AFeC FANDs could be achieved by taking advantage of multi-step tandem endogenous biosynthesis. Specifically, the internalized ALA could firstly bioconvert into PpIX in mitochondria for fluorescence imaging (FLI) and PDT. Subsequently, PpIX-produced heme would individually liberate CO, Fe^2+^, and BV. Among these metabolites, CO could be applied for gas therapy cooperating with PpIX to induce mitochondria damage, and Fe^2+^ could synergize with the released Fe^3+^ for ferroptosis causing membrane disruption. Meanwhile, heme-degraded BV would not only be served as a photothermal therapy (PTT) agent but also enzymatically reduce to antineoplastic BR, which could further collaborate with the released Cur to exert intracytoplasmic chemohyperthermia. Such endogenous multi-metabolite treatment strategy ultimately realizes a continuous and synergistic multi-modality anticancer therapy at different subcellular locations. The present FANDs with admirable API content, on-demand release, and continuous spatiotemporal therapeutic profiles will expand the scope of the development of safe, efficient, and smart nanomedicines for synergistic cancer therapy.

## Results

### Preparation and characterization of the AFeC FANDs

**Fig. 2:**
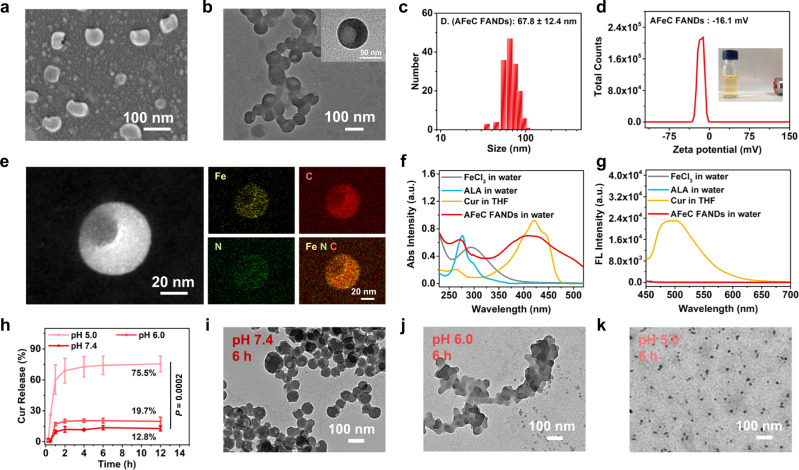
Preparation and characterization of the AFeC FANDs. **a** SEM and **b** TEM images of the AFeC FANDs. **c** Size distribution of the AFeC FANDs. **d** Zeta potential of the AFeC FANDs, inset is their Tyndall effect exhibition. **e** Element mapping for Fe, C, and N of the AFeC FANDs. **f** UV-vis absorption (Abs) and **g** fluorescence (FL) spectra of the free FeCl_3_, 5-aminolevulinic acid (ALA), curcumin (Cur), and AFeC FANDs. **h** In vitro release of Cur from the AFeC FANDs in the buffer solutions of pH 5.0, 6.0 and 7.4. **i**–**k** TEM images of the AFeC FANDs incubated in pH 5.0, 6.0 and 7.4 buffer solutions for 6 h. The results in (**a**, **b**, **e**, **i**–**k**) were representative of three independent experiments. Data in (**h**) were presented as mean ± SD, *n* = 3 independent samples. *P* value was calculated by two-tailed unpaired t-test. Source data were provided in the Source Data file.

The AFeC FANDs were fabricated according to the facile but universal metal coordination-driven self-assembly, in which ALA served as a photodynamic prodrug as well as a metabolic precursor, Fe^3+^ acted as a ferroptosis inducer, and Cur was used as a chemotherapeutic agent. Morphology of the obtained FANDs was investigated by scanning electron microscopy (SEM) and transmission electron microscopy (TEM), manifesting that the AFeC FANDs were bowl-like structures with an average diameter of ~70 nm (Fig. 2a–c). The monodispersed nanosize could be in favor of their tumor accumulation via the enhanced permeability and retention (EPR) effect^42,43^. Zeta potential study revealed that the AFeC FANDs were negatively charged (−16.1 mV) (Fig. 2d). In addition, element mappings monitored by STEM indicated that iron (Fe), carbon (C), and nitrogen (N) elements were homogeneously distributed across the AFeC FANDs, proving the successful formation of the nanostructures from different ingredients (Fig. 2e). To further confirm the existence of different components, we investigated the photophysical features of the AFeC FANDs. As displayed in Fig. 2f, there were apparent absorptions at 271 nm and 428 nm for the AFeC FANDs, which could be attributed to the existing ALA and Cur, respectively. Besides, the free Cur molecule in THF solution displayed intense fluorescence emission at about 510 nm, whereas the fluorescence band was negligible in the AFeC FANDs in aqueous solution due to the aggregation-caused quenching of Cur in the as-fabricated FANDs, further substantiating their exact construction (Fig. 2g)^9,44^. Of particular note, one of the most important advantages of the FANDs was the 100 wt% API content, where the individual drug loading contents of ALA, Fe^3+^, and Cur were assessed to be 77.4%, 2.8%, and 19.8% by high-performance liquid chromatography (HPLC) and standard absorbance curve (Supplementary Fig. 1, 2 and Supplementary Table 1). Such exceptionally high API content provided the promising potential for the clinical translation because of their clear biosafety profile and reduced dose or frequency of administration^9,10^. Furthermore, the AFeC FANDs dispersed in PBS buffers depicted neglectable change during 14 days, and the TEM images at different storage times displayed similar morphology with the freshly prepared FANDs, suggesting their excellent stability under physiological environments (Supplementary Fig. 3, 4). Noteworthily, the metal coordination bond played a key role in the robust colloidal stability, owing to its strength lay between those of common noncovalent interactions and strong covalent interactions, which further synergized with multiple noncovalent interactions including π-π stacking, hydrophobic effect, and hydrogen bonding to enhance the kinetic inertness of the FANDs for prolonged systemic circulation time and augmented tumor accumulation^45–47^. Synchronously, we studied the release of Cur from the AFeC FANDs under different conditions. As shown in Fig. 2h, compared with only 12.8% liberation of Cur under neutral conditions over 12 h, the release of Cur exhibited a slight increase to 19.7% at pH 6.0 and dramatic enhancement to 75.5% at pH 5.0 over the same period of time, which were consistent with the pH-responsive morphological variations of the fabricated FANDs by TEM measurements (Fig. 2i–k). The pH responsiveness may be ascribed to the protonation of the carboxyl groups in acid conditions, cleaving of the metal coordination bonds^48–50^, which would minimize the premature leakage-induced systematic toxicity and concurrently facilitate the precise spatiotemporal therapy at tumor sites.

### Formation mechanisms of the AFeC FANDs

**Fig. 3:**
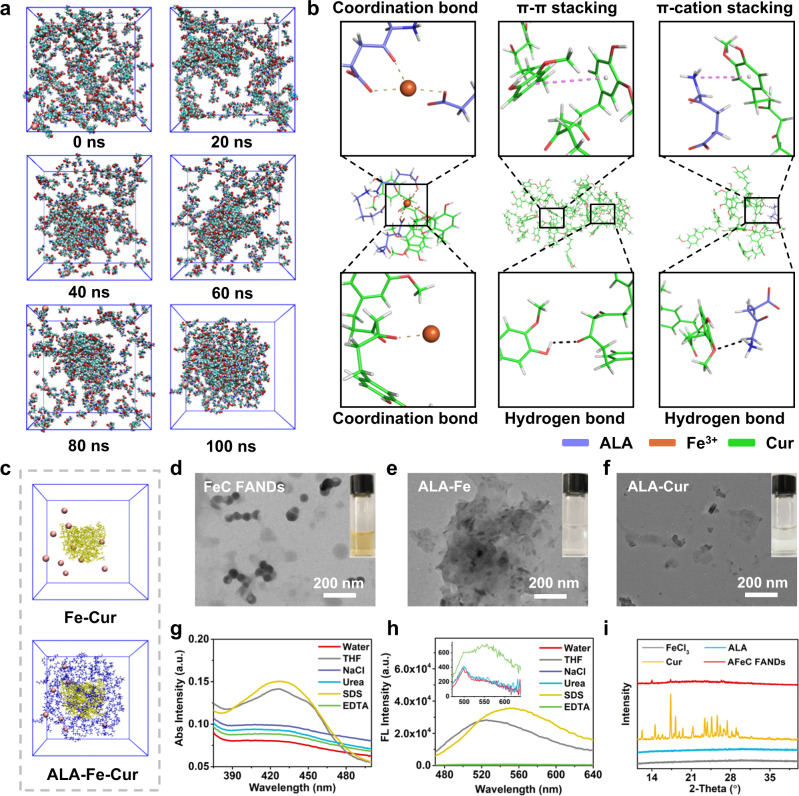
Formation mechanisms of the AFeC FANDs. **a** Molecular cluster changes of 5-aminolevulinic acid (ALA), FeCl_3_, and curcumin (Cur) during 100 ns simulation (blue balls: carbon; deep blue balls: nitrogen; red balls: oxygen; white balls: hydrogen; brown balls: Fe^3+^). **b** Intermolecular interactions analysis in the AFeC FANDs. **c** Evolution of aggregates without or with ALA simulated by molecular dynamics (brown: Fe^3+^; yellow: Cur; blue: ALA). **d**–**f** TEM images of the FeC FANDs, ALA and FeCl_3_ mixture, as well as ALA and Cur mixture. Scale bar: 200 nm. The changes in **g** UV-vis absorption and **h** fluorescence emission of the AFeC FANDs treated with water, THF, NaCl, Urea, sodium dodecyl sulfate (SDS), ethylenediaminetetraacetic acid (EDTA) (V/V = 1:1). **i** XRD pattern of the AFeC FANDs. The results in (**d**–**f**) were representative of three independent experiments. Source data were provided in the Source Data file.

To investigate the possible formation mechanisms of the AFeC FANDs, the molecular dynamics (MD) simulation was performed by the GROMACS program. Figure 3a elaborated the snapshots of the self-assembly process of the AFeC FANDs from molecules to aggregate at different times. Starting from randomly distributed states, the individual molecules gradually assembled into compact architectures along with the time increasing, until t = 100 ns the bulk could be observed explicitly. A more detailed analysis of the driving forces was presented in Fig. 3b, which indicated that the coordination bonds between Fe^3+^ and Cur, as well as the π-π stacking and hydrogen bond between Cur molecules drove the formation of the core, subsequently, ALA was attached to the surface of the core through the coordination bonding with Fe^3+^, and the π-cation interaction and hydrogen bond with Cur. Strikingly, as apparent in Fig. 3c, the ALA molecules (blue) tended to adsorb on the surface of aggregates composed of Fe^3+^ (brown) and Cur molecules (yellow). Beyond that, TEM images described that Fe^3+^ and Cur could obtain bowl-like nanostructures (named as FeC FANDs) using a similar fabrication process, whereas ALA and Fe^3+^, as well as ALA and Cur demonstrated unregular morphology, confirming that the interactions between Fe^3+^ and Cur played a dominant role in the formation of the AFeC FANDs (Fig. 3d–f). To further assess the intermolecular forces in the prepared FANDs, NaCl, Urea, sodium dodecyl sulfate (SDS), and ethylenediaminetetraacetic acid (EDTA) were added to break the electrostatic force, hydrogen bond, hydrophobic interaction, and coordination bonds, respectively. As displayed in Fig. 3g, there was an obvious change in the UV-vis absorption spectrum of the SDS-treated AFeC FANDs, which was similar to that of the AFeC FANDs disassembled in THF. Moreover, with the addition of SDS or EDTA, the self-quenched fluorescence of AFeC FANDs was recovered (Fig. 3h). These phenomena indicated that the AFeC FANDs would dissociate in the presence of SDS or EDTA, suggesting the existence of hydrophobic effects and coordination interaction in the constructed FANDs. Furthermore, according to X-ray diffraction (XRD) analysis, the AFeC FANDs elaborated neglectable diffraction peaks, demonstrating their amorphous structures with low crystallinity (Fig. 3i).

### Biosynthesis based on ALA-PpIX-heme-CO/Fe^2+^/BV-BR metabolism

**Fig. 4:**
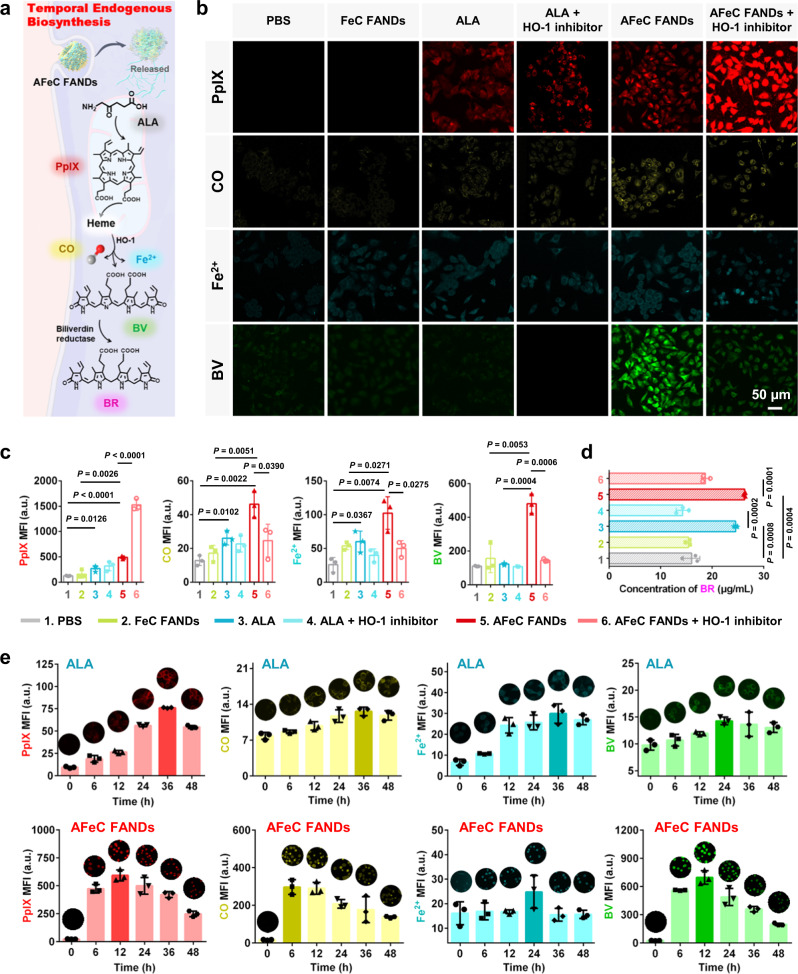
Temporal endogenous biosynthesis of a series of therapeutic metabolites based on ALA-PpIX-heme-CO/Fe^2+^/BV-BR metabolism. **a** Simplified scheme of the tandem metabolism pathway of 5-aminolevulinic acid (ALA). **b** Fluorescence images and **c** the corresponding mean fluorescence intensity (MFI) quantifications of PpIX, CO, Fe^2+^, and biliverdin (BV) in A549 cells with various treatments. **d** Cellular quantitative analysis of endogenous bilirubin (BR) in different groups by ELISA kits. **e** Persistent generation of PpIX, CO, Fe^2+^, and BV in ALA or AFeC FANDs treated A549 cells with different incubation times (The full images of these insets were displayed in Supplementary Fig. 5). Data in (**c**–**e**) were presented as mean ± SD, *n* = 3 biologically independent samples. *P* values were calculated by two-tailed unpaired t-test. Source data were provided in the Source Data file.

After being endocytosed into cancer cells, the AFeC FANDs would disassemble in acid condition, liberating free ALA to produce a series of products with therapeutic potential based on ALA-PpIX-heme-CO/Fe^2+^/BV-BR metabolism. Specifically, ALA would firstly biosynthesized PpIX, and then efficiently converted into heme, which could further endogenously degrade to CO, Fe^2+^, and BV by heme oxygenase-1 (HO-1) catalyzing, where the BV eventually transformed into BR by biliverdin reductase (Fig. 4a)^27,31^.

To clarify the temporal endogenous metabolism of ALA in A549 cells, confocal laser scanning microscopy (CLSM) was employed to investigate the in situ productions of PpIX, CO, Fe^2+^, and BV from different groups (1: PBS, 2: FeC FANDs, 3: ALA, 4: ALA + HO-1 inhibitor, 5: AFeC FANDs, 6: AFeC FANDs + HO-1 inhibitor). The accumulation levels of PpIX, CO, Fe^2+^, and BV under various treatments could be separately detected through monitoring the red fluorescence of PpIX itself, yellow fluorescence of CO probe (FL-CO-1), cyan fluorescence of Fe^2+^ sensor (FeRhoNox-1), and green fluorescence of BV itself. As shown in Fig. 4b, compared to PBS-treated cells, the ALA or AFeC FANDs-incubated A549 cells demonstrated obvious PpIX, CO, and Fe^2+^ signals, affirming the successful endogenous metabolism of ALA. Notably, the BV signal in cells cultured with the AFeC FANDs were much higher than that in the ALA group, which might be attributed to the poor endocytosized of the free ALA molecules. As expected, through introducing HO-1 inhibitors to block heme downstream metabolic pathway, the PpIX accumulation in cells increased while the generations of CO, Fe^2+^, and BV decreased in both ALA-containing groups. Besides, the FeC FANDs-treated cells displayed weaker signals of PpIX, CO, Fe^2+^, and BV than that of the AFeC FANDs group because of the absence of the ALA. The corresponding mean fluorescence intensity (MFI) quantifications demonstrated more details of the metabolite variations in different groups (Fig. 4c). BR production was estimated by using ELISA kits. As depicted in Fig. 4d, different from the PBS and FeC FANDs groups, the ALA or AFeC FANDs-treated cells elaborated apparent BR generation, which would be suppressed with the HO-1 inhibitor addition. This agreed well with the above-mentioned CLSM results.

Moreover, to further confirm the multi-step tandem biotransformation process starting from ALA, we investigated the accumulation of various metabolic components at different times in both ALA alone and AFeC FANDs treated cells (Fig. 4e and Supplementary Fig. 5). With incubation time increasing, the A549 cells treated with ALA alone elucidated significant PpIX, CO, Fe^2+^, and BV generation, even persisting until 48 h. Likewise, apparent temporal formation of multiple pharmaceutical metabolites could be observed in the AFeC FANDs-internalized cells during 2 days. Taken together, these results validated that the exogenous ALA enabled the multi-step tandem biosynthesis of a range of functional metabolites for a sustainable cancer treatment.

### In vitro multi-spatial and multi-model therapy of the AFeC FANDs

**Fig. 5:**
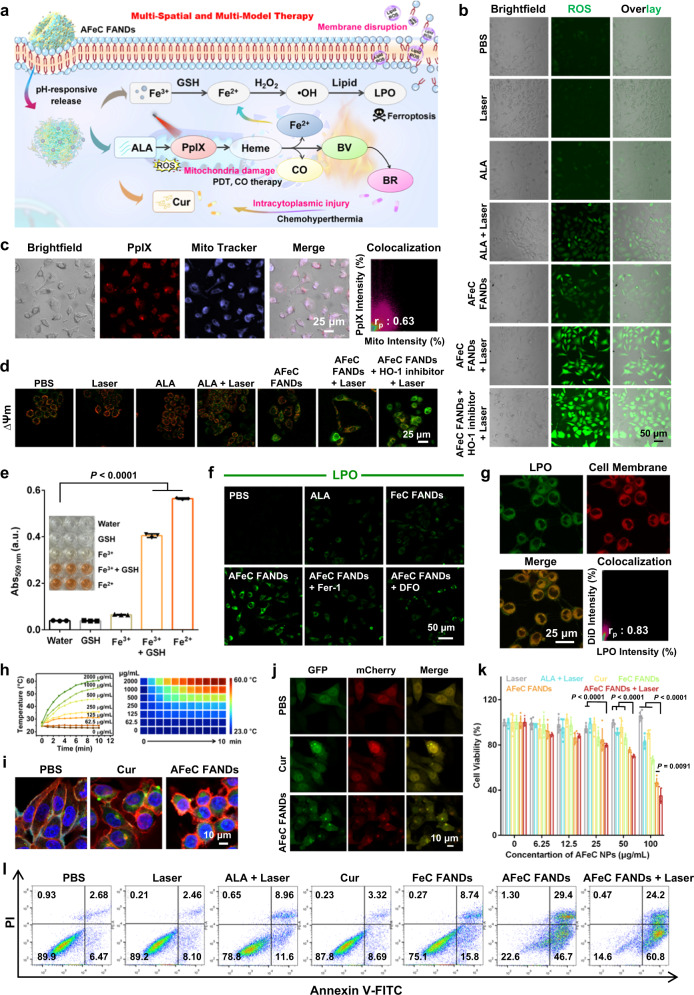
The evaluation of the multi-spatial and multi-model therapy of the AFeC FANDs in vitro. **a** Mechanisms of the AFeC FANDs-mediated continuous spatiotemporal cancer therapeutics. **b** Reactive oxygen species (ROS) generation analysis in A549 cells with different treatments (green: DCFH-DA labeled ROS). **c** Representative mitochondria (Mito) colocalization images of the protoporphyrin IX (PpIX) biosynthesized by AFeC FANDs (r_p_: Pearson’s colocalization coefficient). **d** Mitochondria damage in A549 cells with various treatments detected by JC-1 staining. **e** Absorbance at 509 nm of the complex of Fe^2+^ with 1,10-phenanthroline in different groups. Inset: the corresponding visual color changes. **f** Ferroptosis estimation at cellular levels (green: BODIPY 581/591 C11 labeled lipid peroxidation (LPO)). **g** Cell membrane colocalization assessment of the LPO produced by ferroptosis. **h** Photothermal heating curves of biliverdin (BV) solutions with different concentrations upon 635 nm laser (0.3 W cm^−2^) irradiation, and the corresponding infrared thermal images. **i** Subcellular localization of the curcumin (Cur) in A549 cells (blue: DAPI labeled nucleus, red: DiD labeled cell plasma membrane, green: Cur). **j** Transfection of the Ad-mCherry-GFP-LC3B into A549 cells and measurement of the autophagosomes by CLSM. **k** Cell viabilities of A549 cells with various administrations. **l** Apoptosis study of A549 cells with different treatments by flow cytometric analysis. The results in (**b**–**d**, **f**, **g**, **i**, **j**) were representative of three independent experiments. Data in (**e**, **k**) were presented as mean ± SD, *n* = 3 independent samples in (**e**), *n* = 5 biologically independent samples in (**k**). *P* values were calculated by two-tailed unpaired t-test. Source data were provided in the Source Data file.

In light of the continuous biosynthesis of various ALA metabolites including PpIX, CO, Fe^2+^, BV, and BR, some of which will be further synergic with the Fe^3+^ to initiate ferroptosis as well as Cur-mediated chemotherapy, triggering multi-spatial and multi-model anticancer therapy (Fig. 5a). It has been well reported that the exogenously delivered ALA can specifically biosynthesize photosensitizer PpIX within the mitochondria to perform highly efficient PDT^21–25,51^. As elaborated in Fig. 5b and Supplementary Fig. 6, compared with the neglectable reactive oxygen species (ROS) production in PBS, and only Laser groups, the ALA-treated cells upon laser irradiation (635 nm, 0.3 W cm^−2^) showed increased ROS generation using DCFH-DA as a ROS probe. As expected, when HO-1 inhibitor was added to block the heme downstream metabolism, more ROS generation was detected, due to the elevated PpIX accumulation as previously evidenced in Fig. 4b. Meanwhile, we also investigated the co-localization of PpIX within mitochondria. As described in Fig. 5c, the Mito-Tracker fluorescence showed well overlapped with the red fluorescence of PpIX, indicating that the PpIX was mainly formed in mitochondria. More importantly, apart from PpIX-induced mitochondrial PDT, the heme-liberated CO would also cause oxidative stress-associated mitochondria damage by the elevation of ROS concentration through expediting the second stage of mitochondrial respiration to accelerate oxygen consumption^52–54^. As demonstrated in Fig. 5b, the A549 cells incubated with either ALA or AFeC FANDs without laser irradiation exhibited detectable ROS generation, in large part due to the CO-produced ROS generation. Accordingly, to investigate the collaborative mitochondria collapse, the mitochondrial membrane potential (ΔΨm) was monitored by JC-1 dye, which formed JC-1 aggregates with red fluorescence at high ΔΨm and dispersed as JC-1 monomers with green fluorescence at low ΔΨm. As displayed in Fig. 5d, in comparison to control groups, the AFeC FANDs treated cells without/with photoirradiation depicted decreased red emission and enhanced green signal, confirming the mitochondrial dysfunction caused by CO therapy and PDT.

Beyond that, Fe^3+^ released from AFeC FANDs would be reduced to Fe^2+^ by the over-expressed glutathione (GSH) in tumor cells, which could synergize with the ALA-produced Fe^2+^ to catalyze H_2_O_2_ transform into •OH via Fenton reaction, leading to lipid peroxidation (LPO) and further ferroptosis. As shown in Fig. 5b, the ROS signal of AFeC FANDs treated A549 cells was more significant than that of cells cultured with ALA, which might partly sourced from the Fe^3+^-enhanced •OH production. Furthermore, as displayed in Fig. 5e, similar to Fe^2+^, Fe^3+^ treated with GSH could generate a red-orange complex with 1,10-phenanthroline, indicating GSH reduced Fe^3+^ into Fe^2+^. Subsequently, the •OH formation abilities were investigated through the degradation of methylene blue (MB) in FANDs solution and •OH probe (hydroxyphenyl fluorescein, HPF) in cells. Contrary to the negligible change of MB added with GSH and H_2_O_2_, an apparent absorbance decrease at 663 nm of MB could be observed after further adding Fe^3+^ or AFeC FANDs (Supplementary Fig. 7). Meanwhile, the A549 cells treated with FeCl_3_ or AFeC FANDs validated noticeable •OH generation (Supplementary Fig. 8). After that, BODIPY 581/591 C11 sensor was selectively utilized to evaluate the LPO levels in Fig. 5f and Supplementary Fig. 9, where the AFeC FANDs-incubated cells displayed significant LPO signal. Additionally, with the addition of ferroptosis inhibitor (Ferrostatin-1, Fer-1) or iron chelator (deferoxamine, DFO), the lipid ROS accumulation abated, further proving the ferroptosis induced by AFeC FANDs. Noteworthily, cell membrane colocalization assay testified that the Fe^2+^-initiated LPO mainly existed on plasma membrane (Fig. 5g), showing great potential for realizing specific membrane disruption stemming from the AFeC FANDs.

In addition to mitochondria damage and membrane destruction, the heme-liberated BV could not only be applied as an endogenic photothermal agent for PTT but also enzymatically reduce to antineoplastic BR in cytoplasm, both of which could further collaborate with the released Cur to exert intracytoplasmic thermo-chemotherapy. As presented in Fig. 5h, temperatures of BV solution increased with their concentration and the laser irradiation time, proclaiming the favorable PTT potential of BV for multiple subcellular organelle destruction within cytoplasm. Besides, DAPI staining experiments depicted that BR, as a reduction product of BV, could induce apoptotic body production, leading to cancer cell death (Supplementary Fig. 10, 11). It was reported that Cur could trigger autophagic cell death for tumor suppression^55^. As displayed in Fig. 5i, Cur was mainly released in the cytoplasm, establishing the basis for its contribution to cytoplasmic damage. Additionally, we studied the autophagosome formation in A549 cells by Ad-mCherry-GFP-LC3B. In contrast to the PBS group with diffuse yellow fluorescence, the cells treated with Cur or AFeC FANDs exhibited obvious yellow LC3B dots, verifying the autophagosome generation induced by Cur, thus collaborating with BV and BR to trigger cytoplasmic injury (Fig. 5j). Eventually, the as-designed AFeC FANDs could achieve a continuous and synergistic multi-modality anticancer therapy at different subcellular locations. We thus assessed the antitumor abilities of the AFeC FANDs in vitro using the MTT assay and Annexin V-FITC/PI flow cytometric analysis. As presented in Fig. 5k, cell viabilities of the A549 cells treated with the AFeC FANDs + Laser exhibited the most significant decrease than that of the cells in other groups, which could be ascribed to the continuous spatiotemporal multi-model therapeutic mechanism of the AFeC FANDs. To further estimate the apoptosis, a flow cytometry analysis was performed. Cells treated with ALA under laser irradiation validated certain apoptosis, while free Cur initiated no appreciable apoptosis due to poor water solubility and adverse bioavailability. Meanwhile, outperforming FeC FANDs, the AFeC FANDs and AFeC FANDs + Laser groups manifested the highest apoptotic rates of 76.1% and 85%, respectively, which could be ascribed to the ALA-induced multiple therapeutic metabolite biosynthesis for synergistic cascade tumor therapy (Fig. 5l).

### In vivo antitumor activities of the AFeC FANDs

**Fig. 6:**
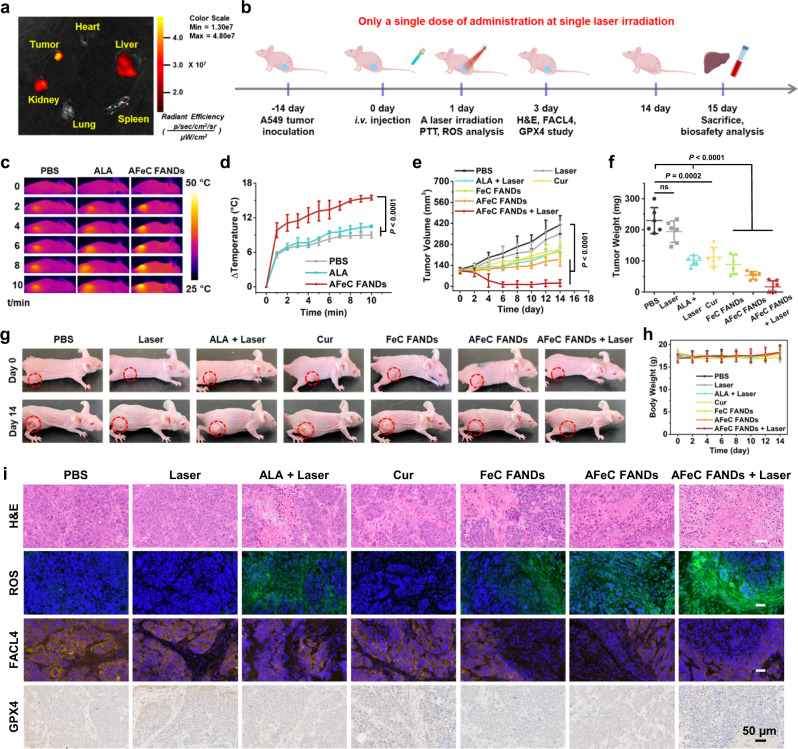
In vivo antitumor activities of the AFeC FANDs. **a** Ex vivo fluorescence imaging of the tumor and major organs from mice after i.v. injection of the AFeC FANDs. **b** Treatment schedule for the anticancer efficiency and mechanism assessments of the as-prepared FANDs. **c** Infrared thermal images of the mice and **d** the corresponding temperature rises of tumors after treatment with PBS, 5-aminolevulinic acid (ALA), AFeC FANDs upon 635 nm laser at different time intervals. **e** Tumor volumes of mice with various treatments during 14 days. **f** Weights of the tumors extracted from mice in varied groups at the end of therapy. **g** Photographs of A549 tumor-bearing mice in different groups before and after 14 day-treatments. **h** Body weights of mice in various groups during 14 day-therapy. **i** Hematoxylin and eosin (H&E), reactive oxygen species (ROS), lipoxygenase enzyme (FACL4), and glutathione peroxidase 4 (GPX4) staining of tumors from mice in different groups. Scale bar: 50 μm. The results in (i) were representative of three independent mice. Data in (**d**–**f**, **h**) were presented as mean ± SD, *n* = 3 biologically independent mice in (d), *n* = 6 biologically independent mice in (**e**, **f**, **h**). *P* values were calculated by two-tailed unpaired t-test. Source data were provided in the Source Data file.

Encouraged by the satisfactory antitumor effects of the AFeC FANDs in vitro, we further investigated their tumor suppression ability in vivo. Initially, biodistribution profiles of the prepared FANDs were assessed via dissecting the tumor and main organs from tumor-bearing mice for fluorescence imaging. As manifested in Fig. 6a and Supplementary Fig. 12, fluorescence signal of the ALA-converted PpIX mainly accumulated in tumor due to the EPR effect, followed by kidney and liver, which could be attributed to the efficient capture of the exogenous FANDs by these main mononuclear phagocyte systems^32^. Subsequently, PTT performance and antitumor efficacy of the FANDs in vivo were carried out on the A549 subcutaneous tumor-bearing mice (Fig. 6b). As revealed in Fig. 6c, d, mice intravenously injected with the AFeC FANDs showed an apparent temperature increase (ΔT = ~15.5 °C) after laser irradiation (635 nm laser, 0.3 W cm^−2^) for 10 min, whereas the ALA and PBS groups under similar conditions only displayed moderate temperature increases, indicating the favorable PTT potency of the AFeC FANDs. In agreement with the cell viability measurements, compared with the rapid tumor growth of PBS and Laser groups, the ALA + Laser, Cur, FeC FANDs, and AFeC FANDs groups described partial tumor growth inhibition (Fig. 6e–g). Intriguingly, the AFeC FANDs-treated mice with photoirradiation validated almost complete tumor eradication. Of particular note, only a single dose of administration at single laser irradiation was required during the whole treatment course owing to the superb full API content of the as-prepared AFeC FANDs, which not only improved their biosafety but also offered promising potential for the clinical translation. Meanwhile, there were no obvious changes in body weight with various treatments for 14 days, which preliminarily verified the reduced health risks of the proposed FANDs (Fig. 6h).

To further investigate the antitumor effects and therapeutic mechanisms, immunostaining assessments were carried out (Fig. 6i). In the hematoxylin and eosin (H&E) staining analysis, it was found that the slice of the AFeC FANDs + Laser group demonstrated the most nuclear pyknosis and cell disruption, because the bluish-purple hematoxylin-stained nucleus shrunk without clear nuclear structure, indicating their salient cancer cell-killing activity. Correspondingly, a significant ROS signal could be observed in the AFeC FANDs + Laser group, which could be attributed to the effective PDT performance and CO therapy. Similarly, substantially decreased lipoxygenase enzyme (FACL4) and glutathione peroxidase 4 (GPX4) expression level further verified the intratumoral ferroptosis induced by the AFeC FANDs with photoirradiation. All the results collectively suggested that the fabricated FANDs displayed maximized antitumor effect because of their desirable API loading, tumor-specific release potency, and continuously multiple therapeutic mechanisms for cancer treatment.

### In vivo safety assessments of the AFeC FANDs

**Fig. 7:**
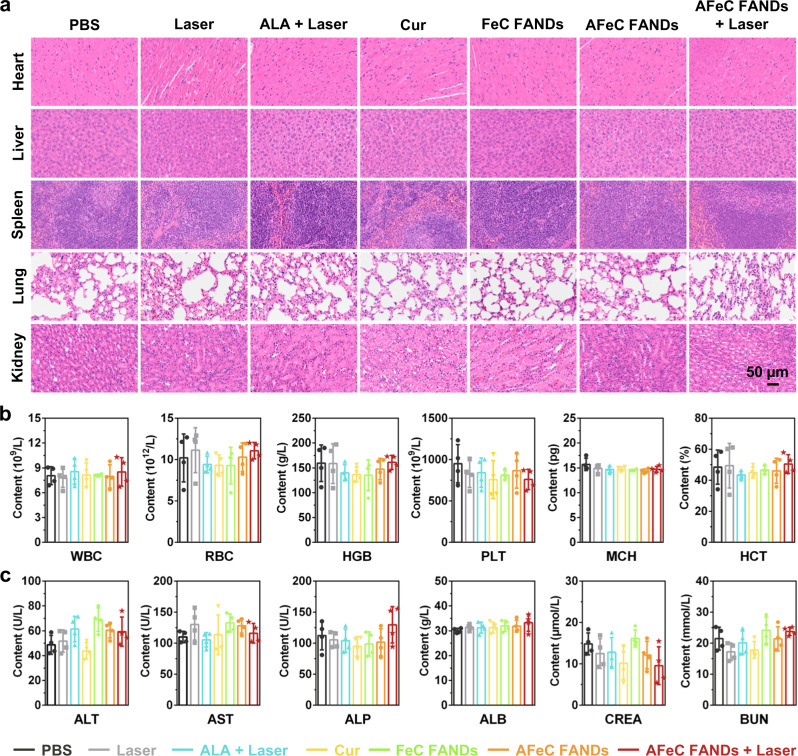
In vivo safety assessments of the AFeC FANDs. **a** Hematoxylin and eosin (H&E) staining of major organs collected from mice after different treatments. **b** The blood parameter and **c** serum biochemistry analysis of mice in various groups. The results in (**a**) were representative of three independent mice. Data in (**b**, **c**) are presented as mean ± SD, *n* = 4 biologically independent mice. Source data are provided in the Source Data file.

Finally, we estimated the biosafety of the AFeC FANDs, which was of importance for clinical translation. After 14-day treatment, major organs (heart, liver, spleen, lung, and kidney) were excised and stained with H&E. As exhibited in Fig. 7a, neglectable pathological changes could be observed in diversified groups, affirming their excellent biocompatibility. Moreover, the blood samples were collected for further hematology analysis, including the blood parameter measurements such as white blood cell count (WBC), red blood cell (RBC), hemoglobin (HGB), blood platelet count (PLT), mean corpuscular hemoglobin (MCH), and hematocrit (HCT) detection, as well as the serum biochemistry measurements as in the case of alanine aminotransferase (ALT), aspartate transaminase (AST), alkaline phosphatase (ALP), albumin (ALB), creatinine (CREA), and blood urea nitrogen (BUN). All of the aforementioned parameters of the mice treated with various formulations illustrated no appreciable variations compared with that of the control group (Fig. 7b, c). All of these results implied that the constructed FANDs showed low systemic toxicity, which may be ascribed to the improved physiological stability, on-demand release profile, and only a single dose as well as single photoirradiation given.

## Discussion

In this work, we developed a full-API nanodrug (FAND) totally consisted of active ingredients (FDA-approved ALA and Cur, as well as human essential Fe^3+^) via metal coordination-driven self-assembly for continuous spatiotemporal cancer therapy. Taking advantage of the synergistic interplay of multiple interactions, the obtained AFeC FANDs demonstrated enhanced stability in physiological environments and stimuli-responsiveness at tumor sites, leading to negligible systemic toxicity and satisfactory biosafety. More importantly, the AFeC FANDs exhibited superior antitumor effect both in vitro and in vivo, owing to their continuous spatiotemporal multi-model antitumor therapeutic mechanism. Specifically, a series of ALA metabolic products with therapeutic potential were endogenously biosynthesized based on ALA-PpIX-heme-CO/Fe^2+^/BV-BR metabolism, which further collaborated with the released Fe^3+^ and Cur for the implementation of multi-modal therapy involving mitochondrial PDT and CO therapy, and ferroptosis-induced membrane disruption, as well as intracytoplasmic thermo-chemotherapy. Finally, a 14-day treatment of the constructed FANDs administrated only a single dose at single laser irradiation showed remarkable therapeutic outcomes and negligible systemic toxicity.

Currently, numerous nanodrugs based on different carriers such as liposomes, polymers, and albumin have been approved for clinical oncology^5,56^. However, there are still considerable challenges restricting their further clinical translation and commercialization (i.e., low API loading content, undesired biodegradability, health risks, and scale-up issues)^57,58^. Recently, carrier-free nanodrugs, mostly consisting of active pharmaceutical ingredients without any inert carrier components, are considered as one of the most attractive candidates to overcome the above-indicated dilemmas, which could effectively avoid the possible long-term toxicity with great clinical promise^59–62^. Noteworthily, in realizing their further practical translation, the compromised colloid stability and unpredictable non-specific release should be vigilant^10,63^. Herein, a concept of full-API nanodrug (FAND, API: ~100 wt%), broadly covering the carrier-free or self-delivered nanodrugs as well as other therapeutic carrier-based nanoformulations is proposed, which can also be equipped with desired physiological stability and site-specific release potency. We have further revealed that the mechanisms of the AFeC FANDs formation were driven by the metal-ligand coordination bond between the Fe^3+^ with Cur and ALA, and the π-π stacking and hydrogen bond between Cur molecules, as well as the π-cation interaction and hydrogen bond of ALA and Cur by molecular dynamic simulation and intermolecular interaction-breaking experiments, endowing the prepared FANDs with favorable stability under physiological environments during 14 days and an enhanced drug release rate of ~60% in acid environments.

In comparison to the conventional carrier-free and other carrier-based nanodrugs, another intriguing property of the as-proposed FANDs was satisfactory safety and patient compliance. Tumor suppression estimation displayed that gratifyingly antineoplastic effects could be realized with a single dose of administration at single laser irradiation. Owing to the 100 wt% API content, enhanced colloidal stability, avoidable premature release, and multi-spatial therapeutic modalities, only a single dose of administration was needed, which could effectively alleviate side effects, as well as greatly improve the biosafety and patient compliance. In addition, thanks to the sufficient API accumulation at the tumor site and multi-model antitumor mechanism, the as-designed FANDs could achieve an efficient PDT outcome with only single laser irradiation while the current clinical phototherapy requires multiple light exposures, thus further making the FANDs with high clinical translational value by reducing the light toxicity and patient tolerance. On these grounds, such simple, safe, and low-cost FANDs based on FDA-approved drugs and human essential elements can not only reveal a high-performance anticancer efficacy through tandem endogenous biosynthesis, but also hold great promise for clinical practice by improving patient compliance.

Looking towards the future, although the constructed FANDs confirmed encouraging characteristics, there are still some challenges that need to be tackled for clinical relevance, for instance, more effective targeting mechanisms should be exploited to improve the tumor accumulation, and more preclinical in vivo toxicity assessments should be systematically applied^64–66^. Despite further optimization that could be done, the smart and efficient full-API nanodrug offers a strategy to broaden the scope of synergistic tumor therapy and facilitate the practical translation of nanodrugs to clinical use.

## Methods

### Ethical statement

All animal experiments were performed in accordance with the guidelines approved by the ethics committee of Beijing Institute of Technology with license number BIT-EC-SCXK (Jing) 2016-0006-M-2020043.

### Materials

Curcumin (Cur) was purchased from Sigma-Aldrich. 5-aminolevulinic acid hydrochloride (ALA·HCl), iron (III) chloride (FeCl_3_), 1,10-phenanthroline, and methylene blue (MB) were purchased from Aladdin. 2,7-dichlorodihydrofluorescein diacetate (DCFH-DA), 3-(4,5-dimethylthiazol-2-yl)-2,5-diphenyltetrazolium bromide (MTT), and 4’,6-diamidino-2-phenylindole dihydrochloride (DAPI) were purchased from Beijing Solarbio Science & Technology. Mito-tracker green, cell plasma membrane staining kit with DiD, and Ad-mCherry-GFP-LC3B were obtained from beyotime. FeRhoNox-1 (Fe^2+^ indicator) and hydroxyphenyl fluorescein (HPF) were purchased from Shanghai Maokang Biotechnology Co., Ltd. Ferrostatin-1 (Fer-1), biliverdin (BV), and bilirubin (BR) were obtained from Shanghai yuanye Bio-Technology Co., Ltd. BODIPY 581/591 C11 was purchased from Cayman Chemical Company. Fetal bovine serum (FBS) was obtained from PAN. Roswell Park Memorial Institute (RPMI) 1640 medium, penicillin-streptomycin, trypsin-EDTA, and PBS (1×, pH 7.4) were purchased from M&C gene Technology (Beijing) Ltd. Anti-Glutathione Peroxidase 4 antibody (ab125066), Anti-FACL4 antibody (ab227256) were purchased from Abcam.

### Preparation of the AFeC FANDs

First, ALA·HCl was dehydrochlorinated into ALA by neutralizing with NaOH to pH = 7.4. Then, the AFeC FANDs were fabricated through the metal coordination-driven self-assembly method through mixing a water solution of ALA·HCl-dehydrochlorinated ALA (1 mM, 1 mL), a water solution of FeCl_3_ (1 mM, 0.05 mL), and a THF solution of Cur (1 mM, 0.2 mL) into distilled water (2 mL), followed by stirring at room temperature for 0.5 h. After that, the obtained FANDs were dialyzed for three days and stored in the dark at 4 °C for further use.

### Characterization of the AFeC FANDs

Morphologies and structures of the AFeC FANDs were measured using the HT-7700 TEM (Hitachi, Janpan) and SU8010 SEM (Hitachi, Janpan). Zeta potential was characterized by DLS (Malvern, UK). EDS pattern was measured by the X-Ray Diffractometer (smartLab SE, Rigaku, Japan). UV-vis absorption feature and fluorescent spectrum were investigated by the UV-1800 spectrophotometer or the SpectraMaxM2/M2e Multi-mode Plate Readers (Molecular Devices, U.S.). Molecular dynamics (MD) simulation was performed by the GROMACS (2019.6) program. The drug loading content (DLC) and drug loading efficiency (DLE) of ALA, Cur, and iron ions in the AFeC FANDs or FeC FANDs were tested using HPLC (1260, Agilent, U.S.), the UV-1800 spectrophotometer, and 1,10-phenanthroline-based UV-vis approach^[1]^, respectively.

1 $${{{{{\rm{DLC}}}}}}=\frac{weight\,of\,loaded\,ALA}{weight\,of\,AF{{{{{\rm{e}}}}}}C\,FANDs}\times 100\%$$

2 $${{{{{\rm{DLE}}}}}}=\frac{weight\,of\,loaded\,ALA}{weight\,of\,total\,ALA\,applied}\times 100\%$$

### In vitro drug release

The release profiles of Cur from the AFeC FANDs were estimated under different pH conditions (7.4, 6.0 and 5.0). Briefly, the AFeC FANDs were added to the buffer solution with various pH values and shaked at 37 °C. The mixture was centrifuged to obtain the supernatant to measure the concentration of the released Cur by UV-vis method at predetermined intervals.

### In vitro evaluation of the endogenous biosynthesis

In this study, the A549 cells from Cell Culture Center (Institute of Basic Medical Sciences, CAMS) were cultured in RPMI 1640 medium containing 10% FBS and 1% penicillin-streptomycin in an incubator with 5% CO_2_ at 37 °C. After seeding A549 cells in the confocal dishes overnight, the cells were treated with PBS, FeC FANDs, ALA, ALA + heme oxygenase-1 (HO-1) inhibitor, AFeC FANDs, AFeC FANDs + HO-1 inhibitor for 24 h. Subsequently, confocal laser scanning microscope (CLSM, A1, Nikon, Japan) (Plan Apo VC 20x, Scanner Zoom: 2.0, PpIX: PMT Offset: 8, Gain: 80, CO: PMT Offset: 0, PMT HV: 65, Fe^2+^: PMT Offset: 0, PMT HV: 80, BV: PMT Offset: 8, PMT HV: 70) were applied to detect the production of PpIX, CO, Fe^2+^, and BV, where the CO and Fe^2+^ were labeled with FL-CO-1^[2,3]^ and FeRhoNox-1^[4]^, respectively. In the work, the fluorescence intensity quantification analyses were performed by the statistical analysis of this CLSM, where the fluorescence intensity value of each pixel point in the image ranged from 0 to 4095, without units. Meanwhile, the BR formation was measured by ELISA kits.

To further estimate the persistence of endogenous metabolism of ALA, A549 cells were cultured in glass-bottom dishes for 12 h. Then, the CO and Fe^2+^ groups were co-incubated with FL-CO-1 or FeRhoNox-1 for another 20 min in the incubator, respectively. Then, fluorescence images and mean fluorescence intensities (recorded as 0 h) were measured by CLSM. After that, the cells were treated with ALA or AFeC FANDs, whose PpIX, CO, Fe^2+^, and BV signals were tested after 6, 12, 24, 36, 48 h by CLSM (Plan Apo VC 20x, Scanner Zoom: 2.5, PMT Offset: 0, PpIX: Gain: 110, CO: Gain: 100, Fe^2+^: Gain: 40, BV: Gain: 100).

### Assessment of ROS generation at cellular levels

A549 cells grew in confocal dishes for 24 h, which were further incubated with PBS, ALA, AFeC FANDs, AFeC FANDs + HO-1 inhibitor at 37 °C for 24 h. Then, 10 μM DCFH-DA were added as a ROS probe for another 20 min incubation. After the photo-excitation (635 nm, 0.3 W cm^−2^, 5 min), the ROS formations were measured through CLSM (Plan Apo VC 20x, Scanner Zoom: 2.0, PMT Offset: 0, Gain: 20).

### Estimation of the co-localization of PpIX with mitochondria

A549 cells were seeded in confocal dishes and cultured overnight. After treating with AFeC FANDs for 24 h, the medium was replaced with Mito-Tracker-containing medium for 20 min co-incubation. Finally, the PpIX fluorescence in mitochondria was measured using CLSM (Plan Apo VC 20x, Scanner Zoom: 3.0, PMT Offset: 0, PpIX: Gain: 100, Mito-Tracker: Gain: 30).

### Extracellular Fe^3+^ reduced by GSH

Typically, FeCl_3_ solution (1 mM, 975 μL) was mixed with GSH solution (10 mM, 20 μL) and incubated for 10 min at 37 °C. Subsequently, after adding 1,10-phenanthroline solution (100 mM, 5 μL), the absorptions at 509 nm of the mixture were recorded.

### Measurement of extracellular •OH

•OH production was studied by MB. Typically, AFeC FANDs were mixed with 10× buffer solution (pH 5.0) and incubated for 30 min. Afterwards, the mixture was added with GSH solution to reduce the released Fe^3+^. After incubation for another 20 min, H_2_O_2_ and MB were added. Finally, the absorption spectrum was measured after 2 h.

### In vitro •OH generation evaluation

A549 cells were cultured in confocal dishes for 24 h, and treated with PBS, FeCl_3_, or AFeC FANDs. After incubation for 12 h, H_2_O_2_ solution (1 mM, 10 μL) was added and cultured for another 12 h. Finally, the •OH signal was labeled with HPF and tested by CLSM (Plan Apo VC 20x, Scanner Zoom: 3.0, PMT Offset: 0, PMT HV: 70).

### In vitro ferroptosis efficacy assay

The LPO was stained with BODIPY 581/591-C11 to assess the ferroptosis effectiveness induced by AFeC FANDs. After incubation for 24 h, A549 cells were treated with PBS, ALA, FeC FANDs, or AFeC FANDs for 24 h. For the AFeC FANDs + Fer-1 and AFeC FANDs + DFO groups, cells were added with Fer-1 or DFO at 12 h post-culture time point of AFeC FANDs. Subsequently, staining the cells with BODIPY 581/591-C11 for 30 min at 37 °C in the dark to obtain the LPO signal in various groups by CLSM (Plan Apo VC 20x, Scanner Zoom: 2.0, PMT Offset: 0, PMT HV: 50).

### Evaluation of the co-localization of LPO with plasma membrane

After culturing overnight, A549 cells were endocytosized with AFeC FANDs for 24 h. After that, the cells were incubated with BODIPY 581/591-C11 and DiD for 20 min to label the LPO and cell membrane. Finally, the co-localization images and Pearson’s colocalization coefficient were recorded by CLSM (Plan Apo VC 20x, Scanner Zoom: 2.5, PMT Offset: 0, BODIPY 581/591-C11: Gain: 50, DiD: Gain: 20).

### Study of the photothermal performance of BV

The BV solution with different concentrations from 0 to 2 mg mL^−1^ was excited by 635 nm laser (0.3 W cm^−2^) for 10 min. Meanwhile, their temperature changes and infrared thermal images were recorded by IR thermal camera every 1 min.

### Detection of the antitumor ability of BR

DAPI staining was utilized to investigate the apoptotic body generation in the cytoplasm. First, A549 cells were inoculated in confocal dishes and cultivated for 24 h. Then, the cells were treated with PBS, BR nanoparticles, ALA, and AFeC FANDs for 24 h. Sequentially, the cells were fixed with 4% paraformaldehyde and stained with DAPI for further CLSM study (Plan Apo VC 20x, Scanner Zoom: 4.0, PMT Offset: 0, PMT HV: 110). Furthermore, the anticancer efficacy of BR was estimated by MTT assay. After cultivating overnight in 96-well plates, the cells were incubated with various BR nanoparticles solution (0, 16.8, 33.6, 67.2, 134.3, and 268.6 μg mL^−1^) for 24 h. Finally, the cell viabilities were detected by the standard MTT assay.

### Investigation of the subcellular localization of Cur

A549 cells were incubated for 24 h and treated with PBS, Cur, and AFeC FANDs for 8 h. After labeling the cell membrane with DiD and stained nucleus with DAPI, then the distributions of Cur in cells were measured by CLSM (Plan Apo VC 20x, Scanner Zoom: 4.0, PMT Offset: 0, DAPI: Gain: 80, Cur: Gain: 110, DiD: Gain: 25).

### Measurement of the autophagosome formation induced by Cur

After cultivating overnight, the A549 cells were transfected with Ad-mCherry-GFP-LC3B for 24 h and further co-incubated with PBS, Cur, and AFeC FANDs for another 24 h. Finally, the autophagosome generation was studied by CLSM (Plan Apo VC 20x, Scanner Zoom: 4.0, PMT Offset: 0, GPF: Gain: 40, mCherry: Gain: 60).

### In vitro cytotoxicity

The cancer-cell-killing abilities of AFeC FANDs were investigated by the MTT assay and flow cytometric analysis. Firstly, the A549 cells were incubated with standard medium in 96-well plates overnight. Then, the cells were treated with PBS, as well as various concentrations of ALA (0, 4.8125, 9.625, 19.25, 38.5, and 77 μg mL^−1^), Cur (0, 1.25, 2.5, 5, 10, and 20 μg mL^−1^), FeC FANDs (0, 1.4375, 2.875, 5.75, 11.5, and 23 μg mL^−1^), and AFeC FANDs solution (0, 6.25, 12.5, 25, 50, and 100 μg mL^−1^) for 12 h incubation. Afterwards, for the laser-involved groups, the cells were irritated with 635 nm (0.3 W cm^−2^, 5 min) laser. After incubation for another 12 h, the cell viabilities were monitored by the standard MTT assay.

For flow cytometry analysis, the A549 cells were seeded in 6-well plates and cultured overnight, then cultivated with PBS, ALA (77 μg mL^−1^), Cur (20 μg mL^−1^), FeC FANDs (23 μg mL^−1^), and AFeC FANDs (100 μg mL^−1^) for 12 h. Subsequently, the irradiation-involved groups were excited with 635 nm laser at 0.3 W cm^−2^ for 5 min. After being incubated for another 12 h, the cells were collected and treated with the Annexin V-FITC/PI Kit, whose results were recorded on a flow cytometry sorter (FACSAria III, BD) and analysed using FlowJo V10. The flow cytometry gating strategy was offered in Supplementary Fig. 13.

### Animals

The 4-6 week-old female BALB/c nude mice were purchased from Peking University Health Science Center. All mice were raised in a 12 h light/dark cycle condition with 23 °C temperature and 55 ± 5% humidity. All mouse experiments were approved by the ethics committee of Beijing Institute of Technology. A549 cell suspensions were subcutaneously injected onto the flank of mice to establish A549 tumor xenograft models.

### Ex vivo biodistribution

At 24 h post-injection AFeC FANDs (1 mg mL^−1^, 200 µL), mice were sacrificed and tumors and major organs (heart, liver, spleen, lung, kidney) were excised, where the fluorescent signals of the biosynthesized PpIX from ALA were measured on the IVIS Spectrum in vivo imaging system (PerkinElmer, U.S.).

### In vivo antitumor therapy

Seven groups of A549-bearing mice (n = 6) were intravenously injected with PBS, ALA (0.77 mg mL^−1^, 200 µL), Cur (0.2 mg mL^−1^, 200 µL), FeC FANDs (0.23 mg mL^−1^, 200 µL), AFeC FANDs (1 mg mL^−1^, 200 µL). Then, the 635 nm laser (0.3 W cm^−2^) was used to irradiate the tumors in the laser-treated groups for 10 min at 24 h-post i.v. injection. During the irradiation process, the tumor surface temperature changes and the corresponding infrared thermal images were recorded by an IR thermal camera. Subsequently, the tumor sizes and body weights of mice with various managements were monitored every 2 days until day 14, where the photos of tumor-bearing mice were taken before and after 14 days.

### In vivo ROS generation ability, ferroptosis effect, and antitumor efficiency study

To further identify the antineoplastic mechanism and performance of AFeC FANDs, the ROS production ability, expressions of FACL4 and GPX4, and the cell disruption of the above-indicated groups were investigated by immunohistochemical and immunofluorescent photomicrographs. For ROS level assay, the mice were intratumorally injected with 10 µL DCFH-DA (10 mM) before photo-excitation. Afterwards, the tumors were excised from the sacrificed mice for immunofluorescent analysis. In addition, the mice with various administration were sacrificed on the 3rd day and the tumors were dissected for staining with FACL4 (Abcam, ab227256, 1:200), GPX4 (Abcam, ab125066, 1:100), or hematoxylin-eosin (H&E), where the FACL4 and GPX4 were used to assess the ferroptosis efficacy, and the H&E stained the nucleus and cytoplasm with blue-purple and red, respectively, to detect the antitumor effect, which were imaged with VS200 research slide scanner (Olympus, Japan).

### Biosecurity estimations

After 14 day-therapy, the mice in diverse groups were sacrificed. Subsequently, the blood samples were collected for blood parameter and serum biochemistry measurements. Along with, the major organs, including heart, liver, spleen, lung, and kidney were collected to stain with H&E for histology studies.

### Statistics and reproducibility

All data were presented as mean ± SD. Statistical analyses were performed by two-tailed t-test using GraphPad Prism 6.01 software. *P* values <0.05 were regarded statistically significant.

### Reporting summary

Further information on research design is available in the Nature Portfolio Reporting Summary linked to this article.

## Supplementary information

Supplementary Information

Reporting Summary

## Data Availability

The data that support the findings of this study are available within the main text and its Supplementary Information file. Source data is provided as Source file. Data is also available from the corresponding author upon request. Source data are provided with this paper.

## Source data

Source Data
