# Supplementary material for: Continuous Spatiotemporal Therapy of A Full-API Nanodrug via Multi-Step Tandem Endogenous Biosynthesis
Source: Nat Commun. 2023 Mar 25;14:1660. doi: 10.1038/s41467-023-37315-0 (PMC10039359; doi:10.1038/s41467-023-37315-0)
Supplement: Supplementary file 1 — Supplementary Information [file 41467_2023_37315_MOESM1_ESM.pdf]

Supplementary information

Continuous Spatiotemporal Therapy of A Full-API Nanodrug via Multi-Step Tandem Endogenous Biosynthesis

Fang Fang[1], Sa Wang[1], Yueyue Song[1], Meng Sun[1], Wen-Cheng Chen[2], Dongxu Zhao[1], Jinfeng Zhang[1,*]

[1]Key Laboratory of Molecular Medicine and Biotherapy, School of Life Science, Beijing Institute of Technology, Beijing 100081, P. R. China.

[2]School of Chemical Engineering and Light Industry, Guangdong University of Technology, Guangzhou 510006, P. R. China

email: jfzhang@bit.edu.cn

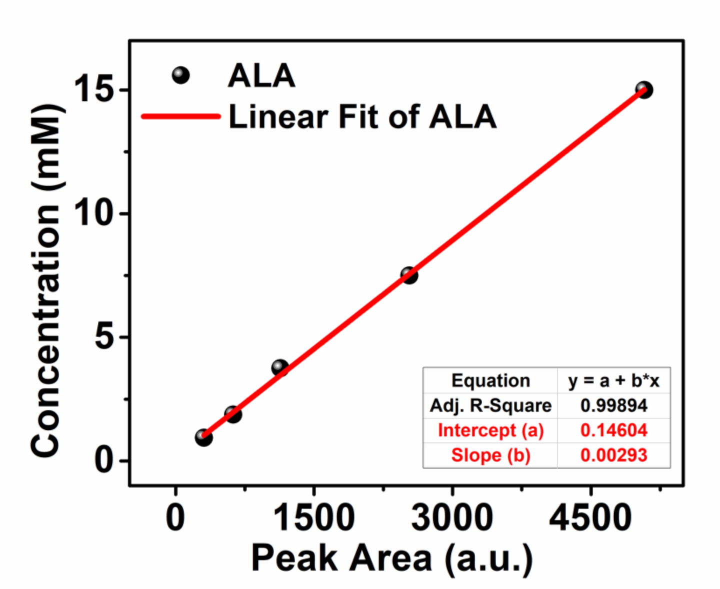

Supplementary Fig. 1 Peak area of 5-aminolevulinic acid (ALA) molecules in water as a function of ALA concentration by high-performance liquid chromatography (HPLC) analysis. Source data were provided in the Source Data file.

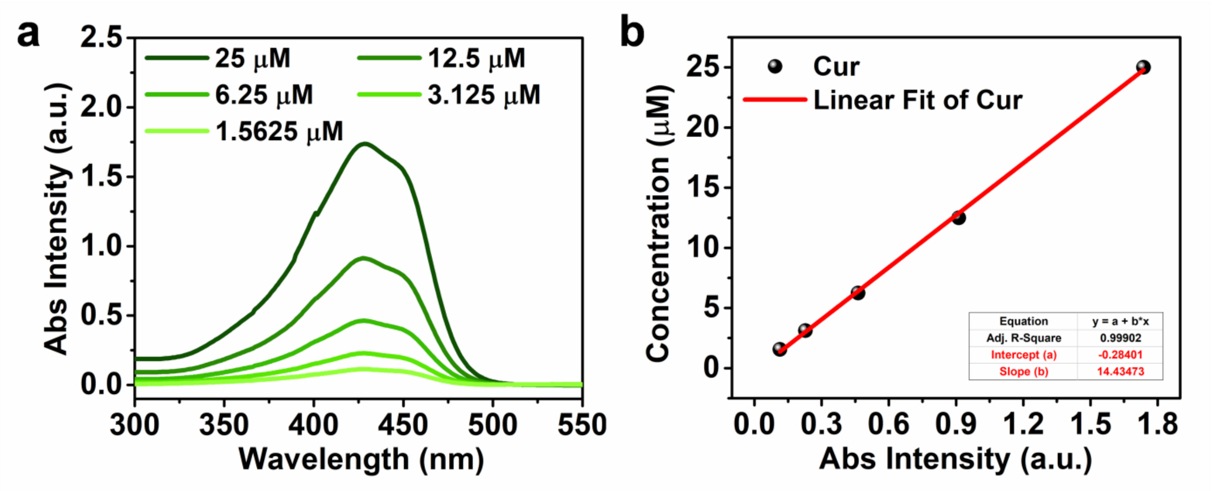

Supplementary Fig. 2 a Standard absorbance curve of curcumin (Cur). **b** Absorbance of Cur molecules at 428 nm (from a mixture of THF and water (V/V = 1:1)) as a function of Cur concentration. Source data were provided in the Source Data file.

Supplementary Table 1. Drug loading contents (DLC) and drug loading efficiencies (DLE) of 5-aminolevulinic acid (ALA), Fe^{3+}, and curcumin (Cur) in the FeC FANDs and the AFeC FANDs.

		FeC FANDs	AFeC FANDs
DLC	ALA	/	77.4 %
	Fe^{3+}	15.1 %	2.8 %
	Cur	84.9 %	19.8 %
DLE	ALA	/	55.6 %
	Fe^{3+}	78.6 %	93.0 %
	Cur	16.8 %	25.4 %

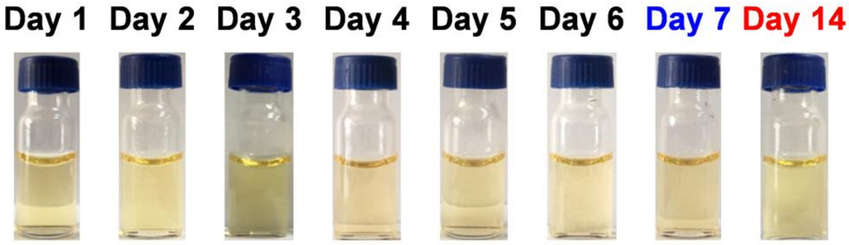

Supplementary Fig. 3 Stability of the AFeC FANDs in PBS buffers for 14 days.

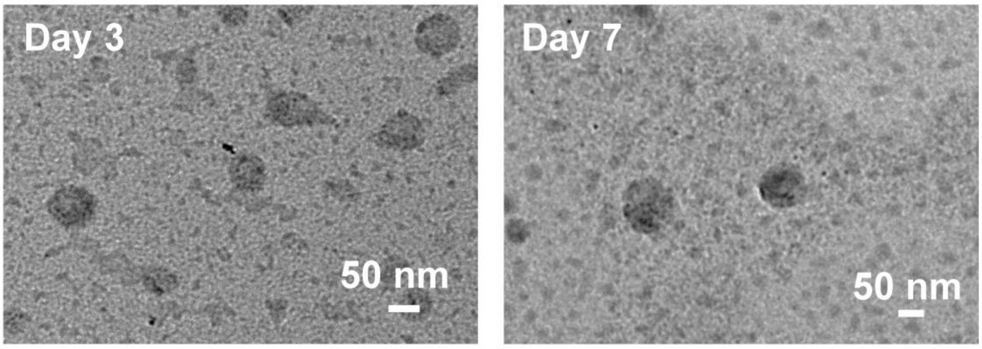

Supplementary Fig. 4 The TEM images of the AFeC FANDs at different storage times. The results were representative of three independent experiments.

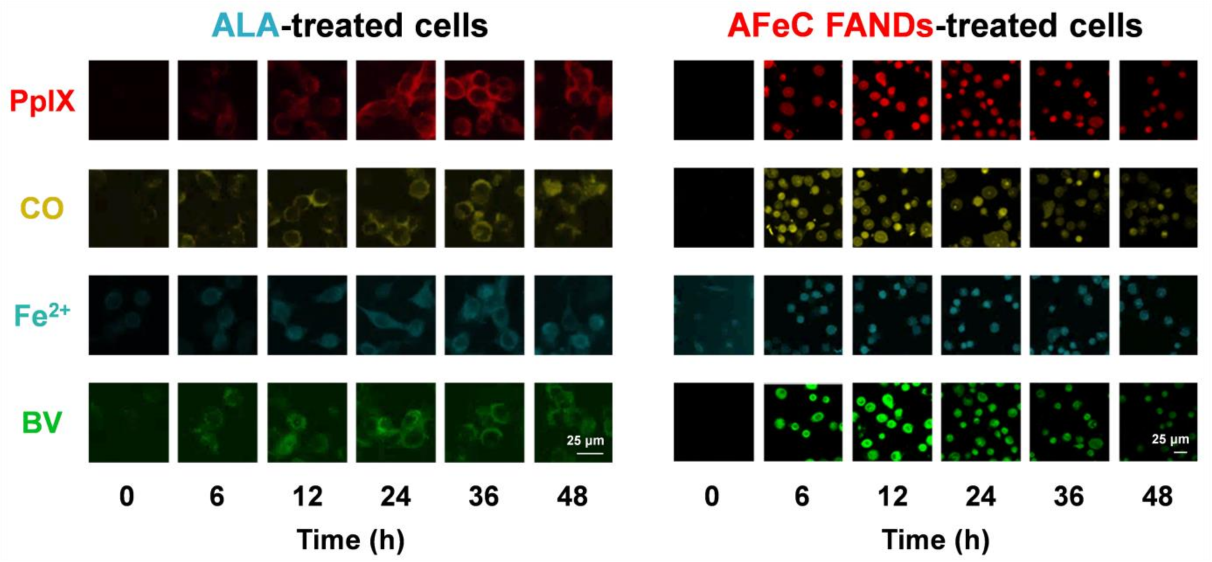

Supplementary Fig. 5 The full images of the insets in Fig. 4e, which were measured by CLSM of the A549 cells treated with ALA or AFeC FANDs at different time to study the PpIX, CO, Fe^{2+}, and BV formation (red: PpIX, yellow: FL-CO-1 labeled CO, cyan: FeRhoNox-1 labeled Fe^{2+}, green: BV).

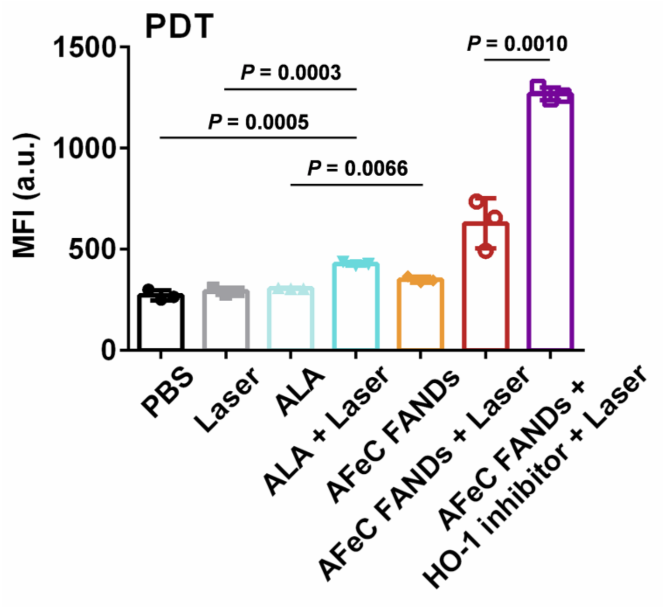

Supplementary Fig. 6 Mean fluorescence intensity (MFI) quantifications of ROS generation in different groups. Data were presented as mean ± SD, n = 3 biologically independent samples. P values were calculated by two-tailed unpaired t-test. Source data were provided in the Source Data file.

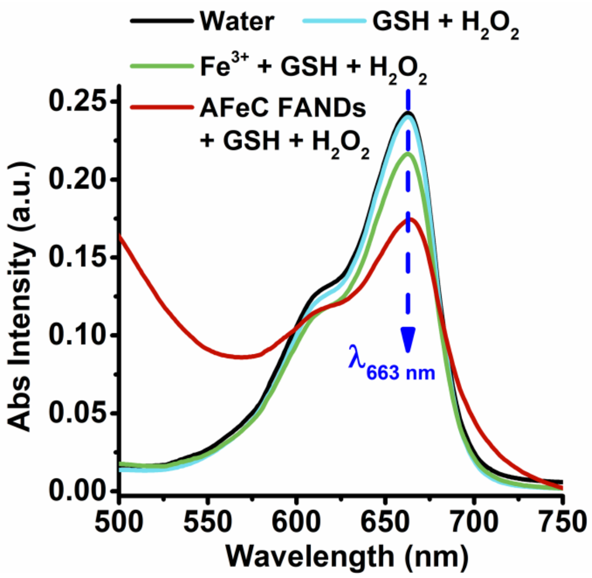

Supplementary Fig. 7 Absorbance of MB in different groups. Source data were provided in the Source Data file.

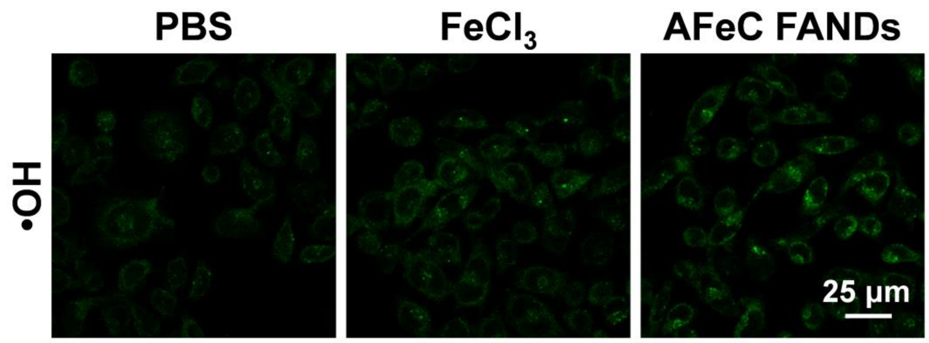

Supplementary Fig. 8 Fe^{3+} induced •OH generation via Fenton reaction measured by hydroxyphenyl fluorescein (HPF). The results were representative of three independent experiments.

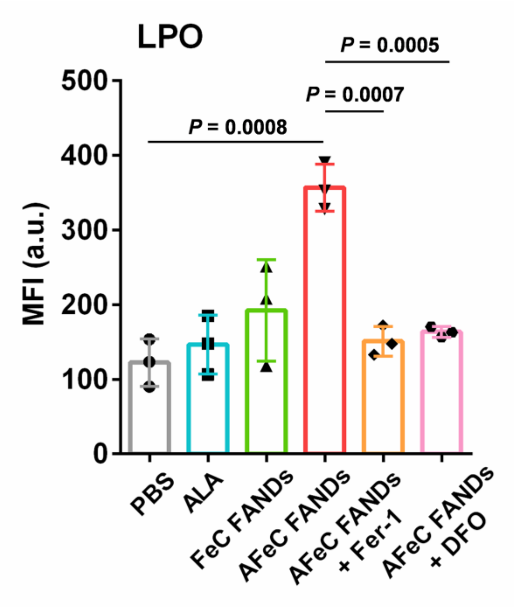

Supplementary Fig. 9 Mean fluorescence intensity (MFI) quantifications of LPO in A549 cells with various treatments. Data were presented as mean ± SD, *n* = 3 biologically independent samples. *P* values were calculated by two-tailed unpaired t-test. Source data were provided in the Source Data file.

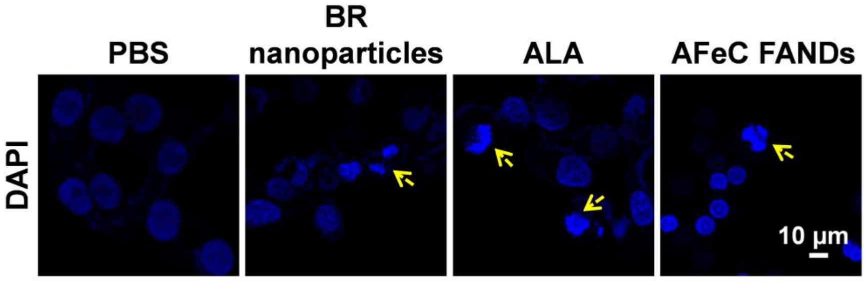

Supplementary Fig. 10 The apoptotic body formation by bilirubin (BR) in A549 cells through DAPI staining. The results were representative of three independent experiments.

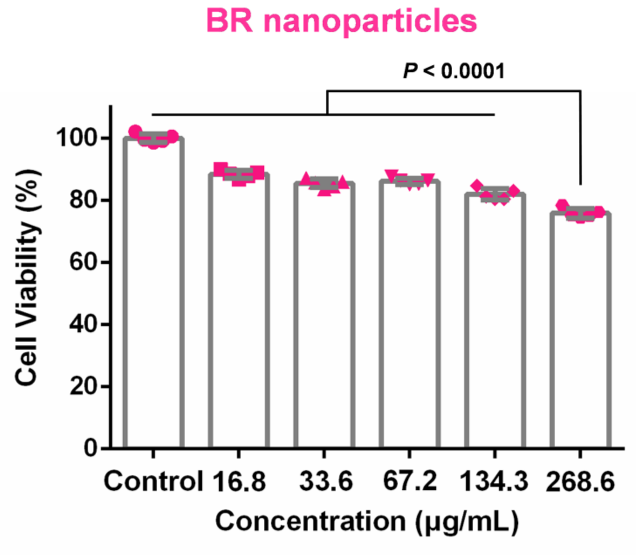

Supplementary Fig. 11 Cell viabilities of A549 cells treated with bilirubin (BR) nanoparticles. Data were presented as mean ± SD, *n* = 5 biologically independent samples. *P* value was calculated by two-tailed unpaired t-test. Source data were provided in the Source Data file.

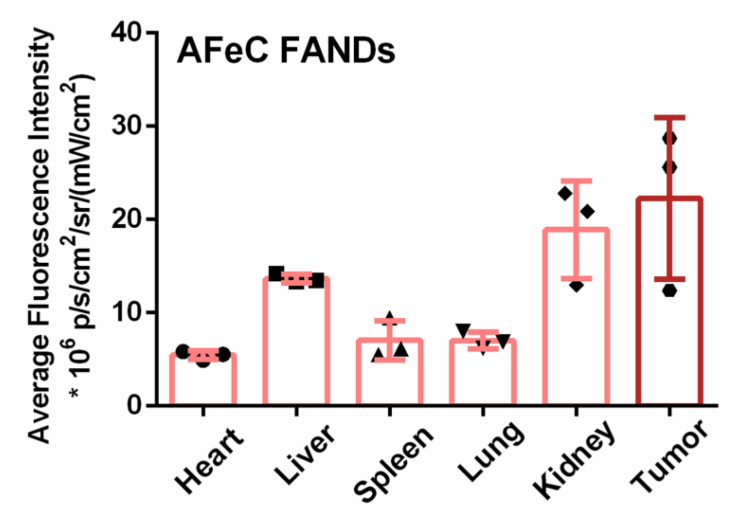

Supplementary Fig. 12 Ex vivo protoporphyrin IX (PpIX) fluorescence (FL) intensities of the tumor and major organs from the AFeC FANDs-treated mice after 24 h. Data were presented as mean ± SD, n = 3 biologically independent mice. Source data were provided in the Source Data file.

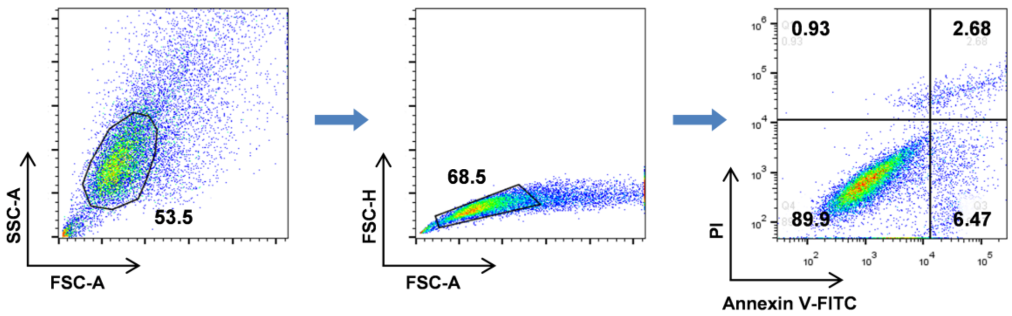

Supplementary Fig. 13 The flow cytometry gating strategy for Fig. 5l.
