# Supplementary material for: Continuous Spatiotemporal Therapy of A Full-API Nanodrug via Multi-Step Tandem Endogenous Biosynthesis
Source: Nat Commun. 2023 Mar 25;14:1660. doi: 10.1038/s41467-023-37315-0 (PMC10039359; doi:10.1038/s41467-023-37315-0)
Supplement: Supplementary file 2 — Reporting Summary [file 41467_2023_37315_MOESM2_ESM.pdf]

nature portfolio

Corresponding author(s): Jinfeng Zhang
Last updated by author(s): Mar 9, 2023

Reporting Summary

Nature Portfolio wishes to improve the reproducibility of the work that we publish. This form provides structure for consistency and transparency in reporting. For further information on Nature Portfolio policies, see our Editorial Policies and the Editorial Policy Checklist.

Statistics

For all statistical analyses, confirm that the following items are present in the figure legend, table legend, main text, or Methods section.

n/a	Confirmed	
☐	☒	The exact sample size (n) for each experimental group/condition, given as a discrete number and unit of measurement
☐	☒	A statement on whether measurements were taken from distinct samples or whether the same sample was measured repeatedly
☐	☒	The statistical test(s) used AND whether they are one- or two-sided<br>*Only common tests should be described solely by name; describe more complex techniques in the Methods section.*
☒	☐	A description of all covariates tested
☐	☒	A description of any assumptions or corrections, such as tests of normality and adjustment for multiple comparisons
☐	☒	A full description of the statistical parameters including central tendency (e.g. means) or other basic estimates (e.g. regression coefficient) AND variation (e.g. standard deviation) or associated estimates of uncertainty (e.g. confidence intervals)
☐	☒	For null hypothesis testing, the test statistic (e.g. F, t, r) with confidence intervals, effect sizes, degrees of freedom and P value noted<br>*Give P values as exact values whenever suitable.*
☒	☐	For Bayesian analysis, information on the choice of priors and Markov chain Monte Carlo settings
☒	☐	For hierarchical and complex designs, identification of the appropriate level for tests and full reporting of outcomes
☐	☒	Estimates of effect sizes (e.g. Cohen's d, Pearson's r), indicating how they were calculated

Our web collection on statistics for biologists contains articles on many of the points above.

Software and code

Policy information about availability of computer code

Data collection	Morphologies and structures of the AFeC FANDs were measured using the HT-7700 TEM (Hitachi, Janpan) and SU8010 SEM (Hitachi, Janpan). Zeta potential was characterized by DLS (Malven, UK). EDS pattern was measured by the X-Ray Diffractometer (smartLab SE, Rigaku, Japan). UV–Vis absorption spectrum and emission spectrum were recorded on the UV-1800 spectrophotometer or the SpectraMaxM2/M2e Multi-mode Plate Readers (Molecular Devices, U.S.). Molecular dynamics (MD) simulation was performed by the GROMACS (2019.6) program. Flow cytometry results were recorded on a flow cytometry sorter (FACSAria III, BD) and analysed using FlowJo V10.
Data analysis	All data were presented as mean ± SD. Statistical analyses were performed by two-tailed t-test using GraphPad Prism 6.01 software. P values < 0.05 were regarded statistically significant.

For manuscripts utilizing custom algorithms or software that are central to the research but not yet described in published literature, software must be made available to editors and reviewers. We strongly encourage code deposition in a community repository (e.g. GitHub). See the Nature Portfolio guidelines for submitting code & software for further information.

Data

Policy information about availability of data

All manuscripts must include a data availability statement. This statement should provide the following information, where applicable:

- Accession codes, unique identifiers, or web links for publicly available datasets
- A description of any restrictions on data availability
- For clinical datasets or third party data, please ensure that the statement adheres to our policy

The data that support the findings of this study are available within the main text and its Supplementary Information file. Source data is provided as Source file. Data is also available from the corresponding author upon request.

Human research participants

Policy information about studies involving human research participants and Sex and Gender in Research.

Reporting on sex and gender	N/A
Population characteristics	N/A
Recruitment	N/A
Ethics oversight	N/A

Note that full information on the approval of the study protocol must also be provided in the manuscript.

Field-specific reporting

Please select the one below that is the best fit for your research. If you are not sure, read the appropriate sections before making your selection.

[x] Life sciences [ ] Behavioural & social sciences [ ] Ecological, evolutionary & environmental sciences

For a reference copy of the document with all sections, see nature.com/documents/nr-reporting-summary-flat.pdf

Life sciences study design

All studies must disclose on these points even when the disclosure is negative.

Sample size	Sample size was determined according the previous experimental experiences and relevant literatures (Bao, W., et al. Nat. Commun. 12, 6399 (2021); Wan Y., et al., Adv. Funct. Mater. 28, 1903436 (2019).). For in vitro, sample sizes were determined for statistical analysis between groups with reliable results. For in vivo, the sample sizes were approved by the ethics committee of Beijing Institute of Technology.
Data exclusions	No data were excluded.
Replication	For ex vivo experiments, individual experiment was independently repeated 3 times with similar results. For murine studies, the reported were pooled results from multiple investigations with similar results or the datadispyayed correspond to a representative assay of at least 3 independent replicates. All the attempts at replication were successful.
Randomization	Mice/cells were randomly allocated into experimental groups.
Blinding	All investigators were blinded to group allocation during experiments.

Reporting for specific materials, systems and methods

We require information from authors about some types of materials, experimental systems and methods used in many studies. Here, indicate whether each material, system or method listed is relevant to your study. If you are not sure if a list item applies to your research, read the appropriate section before selecting a response.

Materials & experimental systems

n/a	Involved in the study
☐	☒ Antibodies
☐	☒ Eukaryotic cell lines
☒	☐ Palaeontology and archaeology
☐	☒ Animals and other organisms
☒	☐ Clinical data
☒	☐ Dual use research of concern

Methods

n/a	Involved in the study
☒	☐ ChIP-seq
☐	☒ Flow cytometry
☒	☐ MRI-based neuroimaging

Antibodies

Antibodies used	Immunohistochemistry:<br>Anti-Glutathione Peroxidase 4 antibody (ab125066, Abcam, 1:100)<br>Anti-FACL4 antibody (ab227256, Abcam, 1:200)
Validation	We relied on publications and on validation sources cited by manufacturer. Links for each antibody are given below:<br>Anti-Glutathione Peroxidase 4 antibody (ab125066, Abcam)<br>https://www.abcam.cn/glutathione-peroxidase-4-antibody-epncir144-ab125066.html<br>Anti-FACL4 antibody (ab227256, Abcam)<br>https://www.abcam.cn/facl4-antibody-ab227256.html

Eukaryotic cell lines

Policy information about cell lines and Sex and Gender in Research

Cell line source(s)	A549 human lung carcinoma cancer cells were purchased from Cell Resource Center, Institute of Basic Medical Sciences, Chinese Academy of Medical Sciences (CAMS).
Authentication	These widely used cell line was authenticated by STR (Short Tandem Repeat) from the commercial sources.
Mycoplasma contamination	The cell line was tested mycoplasma negative by the regular mycoplasma assessment.
Commonly misidentified lines (See ICLAC register)	No commonly misidentified cell line was used in this study.

Animals and other research organisms

Policy information about studies involving animals; ARRIVE guidelines recommended for reporting animal research, and Sex and Gender in Research

Laboratory animals	The 4-6 week-old female BALB/c nude mice were purchased from Peking University Health Science Center. All mice were raised in a 12 h light/dark cycle condition with 23 °C temperature and 55 ± 5% humidity.
Wild animals	No wild animals were involved in this study.
Reporting on sex	The results were independent of sex in this work.
Field-collected samples	No samples were collected from the field in this study.
Ethics oversight	All animal experiments were conducted in accordance with the guidelines approved by the ethics committee of Beijing Institute of Technology with license number BIT-EC-SCXK (Jing) 2016-0006-M-2020043.

Note that full information on the approval of the study protocol must also be provided in the manuscript.

Flow Cytometry

Plots

Confirm that:

☒ The axis labels state the marker and fluorochrome used (e.g. CD4-FITC).

☒ The axis scales are clearly visible. Include numbers along axes only for bottom left plot of group (a 'group' is an analysis of identical markers).

☒ All plots are contour plots with outliers or pseudocolor plots.

☒ A numerical value for number of cells or percentage (with statistics) is provided.

Methodology

Sample preparation	The cells with different treatments were collected, filtered into single cell suspensions, and stained with Annexin V-FITC/PI.
Instrument	BD FACSAria III
Software	FlowJo V10
Cell population abundance	10000 cells were collected from per group for flow cytometry estimation.
Gating strategy	Generally, adhered particles and impurities were gated using FSCA/FSCH approach.

[x] Tick this box to confirm that a figure exemplifying the gating strategy is provided in the Supplementary Information.
